# Estimating Free Energy Barriers for Heterogeneous Catalytic Reactions with Machine Learning Potentials and Umbrella Integration

**DOI:** 10.1021/acs.jctc.3c00541

**Published:** 2023-09-25

**Authors:** Sina Stocker, Hyunwook Jung, Gábor Csányi, C. Franklin Goldsmith, Karsten Reuter, Johannes T. Margraf

**Affiliations:** † Fritz-Haber-Institut der Max-Planck-Gesellschaft, Faradayweg 4-6, 14195 Berlin, Germany; ‡ Engineering Laboratory, University of Cambridge, Cambridge CB2 1PZ, United Kingdom; § School of Engineering, Brown University, Providence, Rhode Island 02912, United States

## Abstract

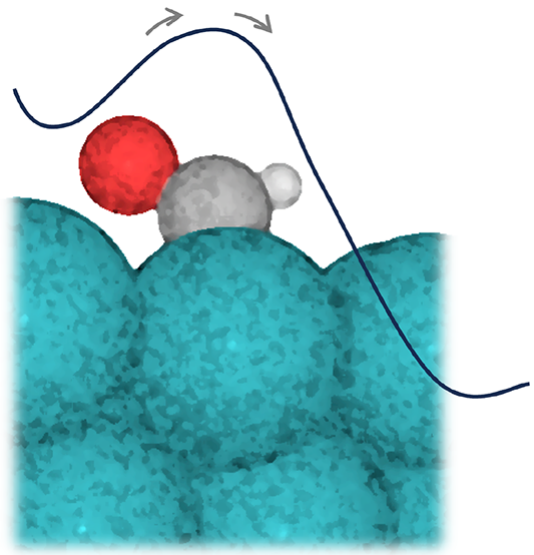

Predicting the rate constants of elementary reaction steps is key for the computational modeling of catalytic processes. Within transition state theory (TST), this requires an accurate estimation of the corresponding free energy barriers. While sophisticated methods for estimating free energy differences exist, these typically require extensive (biased) molecular dynamics simulations that are computationally prohibitive with the first-principles electronic structure methods that are typically used in catalysis research. In this contribution, we show that machine-learning (ML) interatomic potentials can be trained in an automated iterative workflow to perform such free energy calculations at a much reduced computational cost as compared to a direct density functional theory (DFT) based evaluation. For the decomposition of CHO on Rh(111), we find that thermal effects are substantial and lead to a decrease in the free energy barrier, which can be vanishingly small, depending on the DFT functional used. This is in stark contrast to previously reported estimates based on a harmonic TST approximation, which predicted an increase in the barrier at elevated temperatures. Since CHO is the reactant of the putative rate limiting reaction step in syngas conversion on Rh(111) and essential for the selectivity toward oxygenates containing multiple carbon atoms (C_2+_ oxygenates), our results call into question the reported mechanism established by microkinetic models.

## I Introduction

It is well-known that the rate, selectivity, and yield of a chemical process can be controlled by the use of catalysts.^1^ In simple terms, this works via a modulation of the relative energetics of the reaction intermediates and transition states, which partake in the overall process leading from the reactants to the products. Here, the energetic barriers for elementary reaction steps are of particular interest, since (according to transition state theory, TST) the rate constant of each step is proportional to the exponential of the free energy barrier.^2,3^ The accurate computational prediction of free energy barriers for elementary reactions is thus essential for understanding the detailed mechanisms of catalytic processes and for designing new catalysts.^4−6^

Predicting free energy barriers accurately is notoriously difficult, however. This is because the simple picture of the barrier as an energy difference between a single minimum configuration and a transition state does not apply at elevated temperatures. Instead, a rigorous free energy calculation requires extensive sampling of the configuration space along a suitable reaction coordinate, *e.g*., via Transition Path Sampling,^7^ Metadynamics,^8^ or Umbrella Sampling.^9,10^

These methods are commonly applied in biomolecular simulations, *e.g*., to study the binding affinities of drug candidates to certain enzymes. Here, computationally efficient empirical force fields are available so that extensive sampling is not an insurmountable issue. Unfortunately, this is not the case in heterogeneous catalysis, where the surface of a solid catalyst must be accurately modeled. This requires the use of computationally expensive first-principles methods like density functional theory (DFT) with the consequence that rigorous free energy calculations are rarely performed in this context.

To avoid this computational bottleneck, DFT based catalysis research instead typically relies on a more approximate treatment of free energy barriers. In the simplest case, temperature effects on the barrier are simply neglected so that the Helmholtz free energy surface (FES) is approximated by the 0 K potential energy surface (PES). This simplifies the problem to finding the transition state between the initial and final minimum configuration of the reaction on the PES, which can be achieved, *e.g.*, with the Climbing Image Nudged Elastic Band (CI-NEB) approach and related methods.^11^

Additionally, free energy corrections to the PES barrier can be computed. These rely on calculating the vibrational modes of the reactants in the initial, final, and transition state configurations and are thus somewhat more involved.^12^ Most commonly, the harmonic approximation (HA) is used in this context, meaning that a second-order Taylor expansion of the PES around the configurations of interest is performed, from which the finite-temperature free energy corrections can be calculated analytically. While applying the HA is relatively straightforward in principle, it is known to be inadequate at higher temperatures and for low-frequency modes, such as hindered translations or rotations.^12,13^ These factors lead to some ambiguity on how to treat (or whether to neglect) slow modes and overall add to the uncertainty of predicted free energy barriers.^14^

Fortunately, the success of machine-learning (ML) interatomic potentials in chemistry and materials science in recent years has opened a new perspective for free energy calculations in heterogeneous catalysis. Machine-learning potentials can provide fast and accurate surrogate models of the DFT PES,^15−17^ so that the kind of extensive sampling that is required for rigorous free energy calculations becomes feasible.

**Figure 1:**
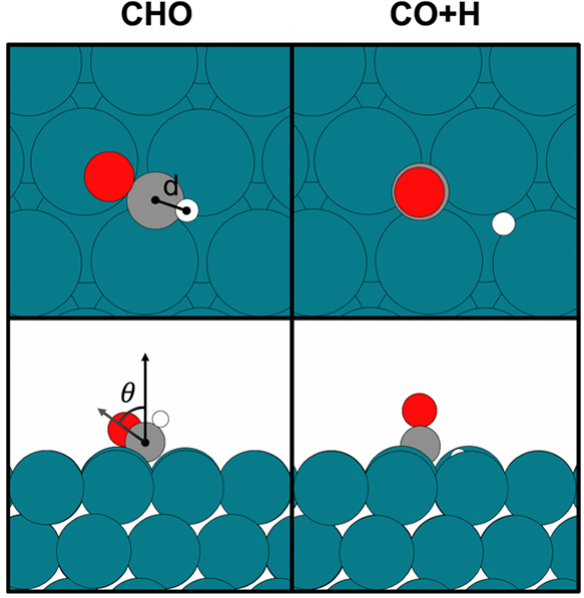
Top and side views of local minimum configurations for CHO and CO+H on the Rh(111) surface. The geometric measures *d* and θ, which define the collective variable used below, are indicated for CHO.

In this contribution, we demonstrate this for the initial hydrogenation step of CO on Rh(111), focusing on the kinetic stability of the CHO reaction intermediate (Figure 1). This system is of particular interest since Rh-based catalysts are unique in their ability to convert syngas (CO and H_2_) to higher oxygenates like ethanol and acetaldehyde with appreciable selectivity.^18−21^ We focus on CHO because it is the reactant in the putative rate limiting step for ethanol and acetaldehyde synthesis, according to some microkinetic models.^19^ Furthermore, the relative formation rates of CHO and COH ultimately determine the selectivity for the C_2+_ oxygenates.

We present a workflow that combines the iterative training of Gaussian Approximation Potentials (GAP) with the Umbrella Integration (UI) approach, yielding a fast and accurate method for free energy calculations in surface catalysis. Subsequently, the results are compared to previously reported harmonic estimates of the free energy barrier, and the influence of different density functional approximations is discussed.

## II Methods

### II.1 Umbrella Integration

The UI approach is a hybrid between two popular free energy methods, namely, Umbrella Sampling (US) and Thermodynamic Integration.^10,22,23^ As in the US,^9^ a set of harmonic biasing potentials that span the scope of a collective variable (CV) ξ are defined:
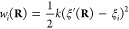
1Here, *w*_*i*_ is the *i*th biasing potential with a spring constant *k*, which restrains the sampling around the window center *ξ*_*i*_. ξ′(**R**) is a function that maps the coordinates of system **R** to the collective variable ξ, which can be an interatomic distance, angle, coordination number, or more complex function of the atomic coordinates. Note that ξ can, in principle, be higher dimensional, although we only consider the 1D case herein. The biasing potential restrains simulations to a region in phase space close to *ξ*_*i*_, which is referred to as the *i*th window.

With these potentials, a series of biased molecular dynamics (MD) simulations are performed, where the biases ensure that energetically unfavorable parts of the PES are sampled sufficiently. In conventional US, the FES along ξ is then typically obtained from the corresponding MD ensembles using the weighted histogram analysis method (WHAM).^24−26^ In UI, the FES is instead obtained by estimating the gradients of the free energy *F* with respect to ξ.^10,22,23^ To this end, one merely needs the mean value of ξ (*ξ̅*_*i*_) for each biased ensemble:
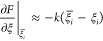
2This approximation holds under the assumption that the distribution in ξ is unimodal and symmetric, which is generally the case if *k* is sufficiently large, so the main remaining source of uncertainty is the statistical error on *ξ̅*_*i*_. The FES can then be obtained by integrating . Stecher et al. showed that this can be done in an uncertainty aware fashion using Gaussian Process Regression (GPR) and we follow this approach herein.^27^ All UI simulations below are performed with the atomic simulation environment (ASE) and a Langevin thermostat.^28^

### II.2 Gaussian Approximation Potentials

GAP models are a well established class of ML interatomic potentials that are also based on GPR.^29^ Since the GAP approach was extensively reviewed recently, we only briefly summarize the details of the potential used herein.^15^ All hyperparameters as well as the potential itself are provided as Supporting Information in this article.

We express the total formation energy of the surface–adsorbate system in terms of a two-body and a many-body contribution, with the latter being described by the Superposition of Atomic Densities (SOAP) representation.^30^ The potential is trained in an iterative fashion by running UI simulations and NEB calculations to generate new configurations with the GAP model and augmenting the training set accordingly (see below for details). This provides a data-efficient and automated workflow for generating a training set that covers the relevant parts of the phase space. To avoid redundancies in the configurations that are added to the training set, these are selected according to the kernel distance between new configurations and the current training set (so-called farthest point sampling, FPS).^31^

### II.3 Density Functional Theory

In computational catalysis at metal surfaces, semilocal functionals based on the Generalized Gradient Approximation (GGA) are most commonly used. This is due to their relatively high computational efficiency compared to those of hybrid DFT and explicit many-body methods. Additionally, these functionals are fairly accurate in describing the energetics of molecular adsorbates on transition metal surfaces, particularly when combined with appropriate dispersion corrections.^32,33^

To explore the influence of different computational setups, two separate GAP models were trained with different reference data. On one hand, the revPBE^34^ functional and vdW^surf^ dispersion correction^35^ were used, as implemented in the full-potential numerical atomic orbital code FHI-aims.^36^ Here, light integration settings and a tier-1 basis set were used, as is usually done for *ab initio* MD simulations. On the other hand, the BEEF-vdW^37^ functional was used, as implemented in the plane-wave code QuantumEspresso, using ultrasoft pseudopotentials and a kinetic energy cutoff of 500 eV for orbitals and 5000 eV for the density.^38^ Note that the revPBE setup was used in the iterative training scheme, while the BEEF-vdW potential was subsequently trained in the same configurations. In the following, all figures show revPBE-based results unless otherwise noted. All simulations were performed in a 3 × 3 × 4 Rh(111) surface slab (where the lower two slab layers were constrained during all simulations). A 4 × 4 × 1 *k*-grid was used to sample the Brillouin zone.

For harmonic free energy corrections, vibrational frequencies were obtained at the DFT level via finite-difference Hessians of the adsorbates in the initial and transition state geometries. Since vibrational frequencies are more sensitive to the convergence of geometry optimizations than total energies, ground state geometries were tightly converged to maximum force norms of 0.01 eV/Å. The CI-NEB transition state geometries were refined using the iterative Hessian diagonalization algorithm implemented in the Sella package.^39^

As is common practice in computational catalysis, the vibrational frequencies were computed by generating the finite differences of the Hessian for the adsorbate atoms only. This was done to ensure consistency with the literature, in particular ref (19). For the UI calculations, only the lower two layers of the metal slab were constrained. This introduces a slight inconsistency between the UI and the HA calculations. To evaluate the effect of this inconsistency, vibrational calculations were repeated under inclusion of the top two metal layers for the BEEF-vdW functional. From this, we find that including these atoms in the Hessian only has a marginal effect on the harmonic free energy barrier, which increases by 0.01 eV.

## III Results

### III.1 Minimum Energy Path and Collective Variable

The focus of this work will be on the first hydrogenation step of CO on Rh(111) to form CHO (Figure 1). Here, the small barrier for the reverse reaction is of particular interest, since it determines the stability of the CHO intermediate on the surface. The geometric changes between the reactant and product can be described in terms of the angle between the CO bond and the surface normal (θ, in Radians) and the C–H distance (*d*, in Å). While these parameters would in principle form adequate CVs for this reaction, the computational effort for free energy calculations rises substantially with each dimension that is considered. We therefore first define an effective one-dimensional CV.

**Figure 2:**
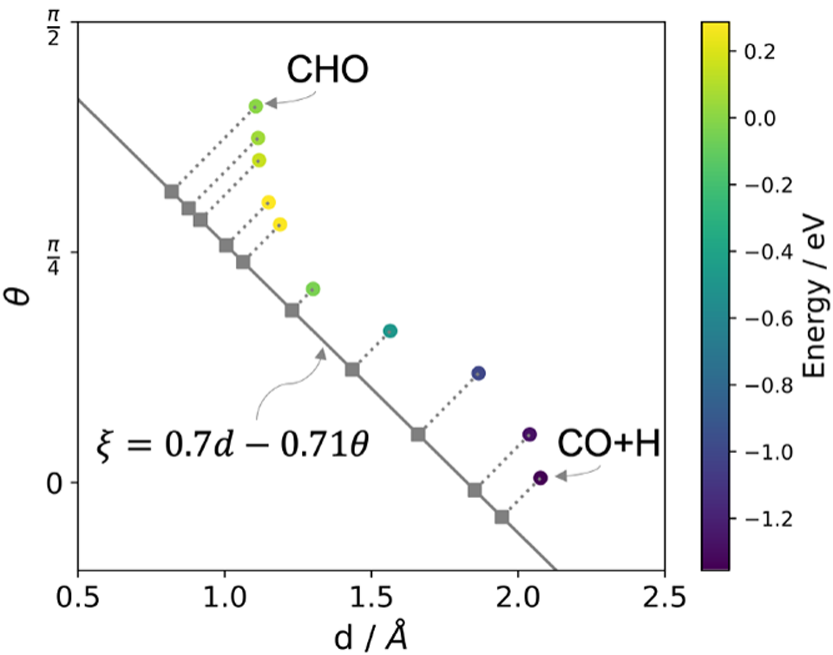
Nudged elastic band images plotted in terms of the angle between the CO and the surface normal (θ) and the C–H distance (*d*). The color bar shows the relative energies of the images in eV, with the yellow points being close to the transition state. The collective variable used herein is a linear combination of θ and *d*, indicated by the gray line.

To this end, we take advantage of the fact that the CI-NEB method allows calculating minimum energy paths for chemical reactions on the PES. This yields a series of configurations (termed images) that connect reactant and product and include the transition state. In Figure 2, the NEB images of CHO formation are plotted with respect to *d* and θ. This reveals that the reaction first proceeds by a gradual decrease of *d* and increase of θ, until the transition state is reached. Subsequently, θ further increases, until the product geometry is obtained.

Based on this path, we define the CV ξ used in the following as a linear combination of *d* and θ, connecting the reactant minimum configuration and the transition state. Here, the units of the parameters are chosen to render the overall CV unit-less. This CV is plotted as a gray line in Figure 2, with the projection of each NEB image indicated by the dotted lines and diamonds. Note that any parallel line in this plot effectively corresponds to the same CV. This shows that all NEB images are well separated on this scale, indicating that ξ is a suitable reaction coordinate. For the following free energy calculations, 50 evenly spaced biasing potentials are defined in the range ξ = −0.2–0.85, with a spring constant *k* = 50 eV.

### III.2 Potential Training and Validation

The GAP potential used to run the UI simulations is trained in an iterative fashion. Specifically, we define an initial training set of 50 configurations, consisting of the images from the DFT based NEB calculation shown in Figure 2, dimer curves of the light element pairs (CO, CC, OO, HH, OH, and CH), as well as optimized and rattled configurations of the pristine surface. This set is used to train an initial, coarse GAP model.

While far from chemically accurate, this potential can directly be used to run biased MD simulations at 573 K in order to explore the phase space relevant to the UI simulations. As there is some redundancy between neighboring windows, we randomly select ten windows from which to sample from. Subsequently, a diverse set of ten structures is extracted from these configurations via FPS and evaluated with single-point DFT calculations. This data is used to validate the accuracy of the current potential and added to the training set for the next iteration.

**Figure 3:**
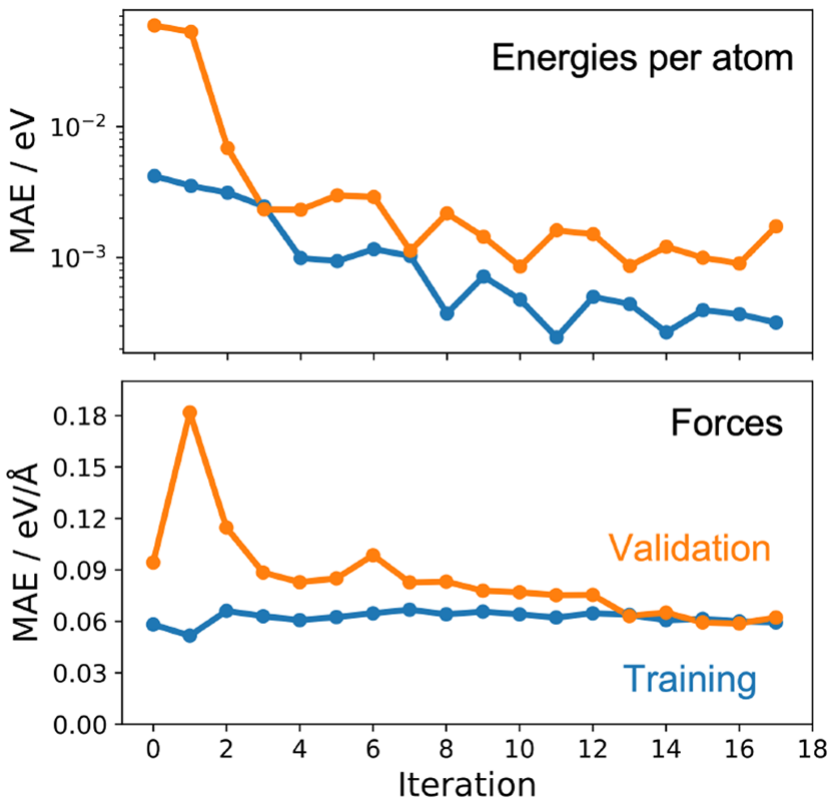
Evolution of mean absolute errors (MAE) on energies (top) and forces (bottom) during iterative training. At each iteration, new configurations are generated via Umbrella Sampling and are used as a validation set. These structures are added to the training set for the next iteration.

We find that energies and forces are well converged in 17 iterations, with mean energy errors well below 10 meV per atom and force errors below 75 meV/Å. Note that after initial convergence was observed in iteration 12, the potentials are additionally used to perform NEB calculations to ensure that the minimum energy paths of the GAP and the underlying DFT functional coincide. The evolution of the corresponding training and validation errors is shown in Figure 3. Importantly, the validation errors are for unseen configurations from the exact kind of simulation that we intend to run with this model. This gives us high confidence that the potential will be sufficiently accurate and stable for this purpose.^40^

As indicated above, the energies and forces on the final training set were additionally recalculated at the BEEF-vdW level. This data was used to train a second potential without performing additional training iterations. This allows us to explore the impact of using different computational setups for the free energy calculations.

### III.3 Free Energy Calculations

**Figure 4:**
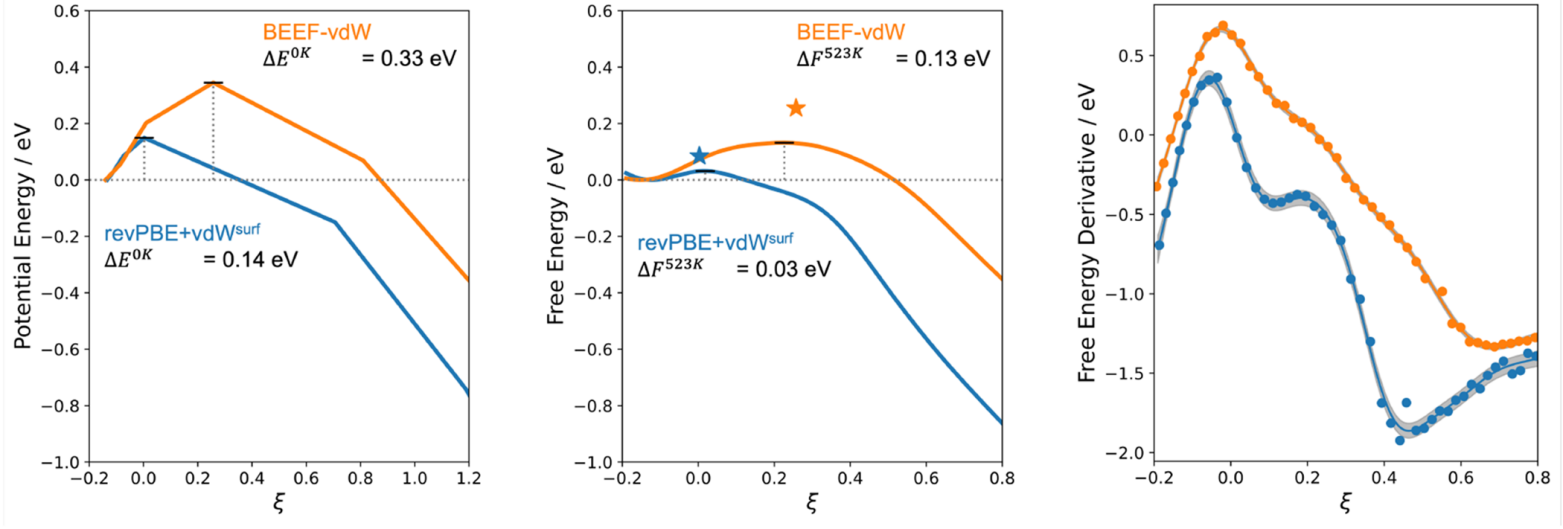
Potential energy surface from Nudged Elastic Band calculations (left) and free energy surface from Umbrella Integration (center). Umbrella Integration estimates the relative free energies from the derivative of the free energy with respect to the collective variable (right). The estimated barriers using harmonic free energy corrections are indicated by the stars in the central plot. Calculations were performed with GAP models trained on the revPBE+vdW^surf^ and BEEF+vdW data, respectively. The gray region in the rightmost plot indicates the standard deviation of the Gaussian Process model used for Umbrella Integration.

The converged potentials were used to run extensive US calculations with 100 ps trajectories per window, at 523 K, which is the experimental operating temperature in ref (19). This amounts to 16 ns of dynamics overall, underscoring why this type of simulation is computationally prohibitive at the first-principles level. The resulting FESs at the revPBE+vdW^surf^ and BEEF+vdW levels are shown in Figure 4, along with the PESs obtained with NEB calculations.

A comparison of the PESs and FESs reveals that thermal effects decrease the barrier for decomposition of CHO significantly. Indeed, the barrier nearly vanishes at the revPBE+vdW^surf^ level, indicating that CHO is not a stable intermediate at 523 K at all. At the BEEF-vdW level, a small barrier of 0.13 eV remains. Figure 4 also shows the free energy derivatives, as defined in eq 2. This shows that the GAP-based MD simulations provide good coverage of the CV range. More importantly, the derivatives display no discontinuities, which would point to lack of convergence or broken ergodicity.

### III.4 Rate Constants

For catalytic applications, the free energy barrier is mainly of interest as an intermediate quantity for computing rate constant *k*. The most common framework for computing *k* from first-principles is Transition State Theory (TST). In this context, the rate constant is defined via the Eyring equation as
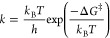
3where Δ*G* is the free energy barrier. One can illustrate the importance of thermal effects on rates by plugging in the potential energy barrier Δ*E* instead of the free energy barrier. At the BEEF-vdW level the corresponding rates differ by a factor of 85, i.e., by almost 2 orders of magnitude.

Equivalently, the Eyring equation can also be formulated in terms of the partition functions of the initial and transition states, *Q*_IS_ and *Q*_TS_:
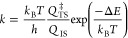
4This equation is the basis of the commonly used Harmonic Approximation (HA) for computing rate constants. This assumes that the adsorbate is tightly enough bound to the surface so that all degrees of freedom can be described as vibrations. In this case, the corresponding partition functions can be computed from the vibrational modes of the adsorbate in the initial and transition state configurations. Depending on whether the vibrations are described as classical or quantum harmonic oscillators, the corresponding expressions are
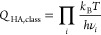
5and
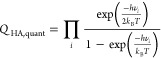
6where the product runs over all 3*N* vibrational modes and *ν*_*i*_ is the frequency of mode *i*.

**Figure 5:**
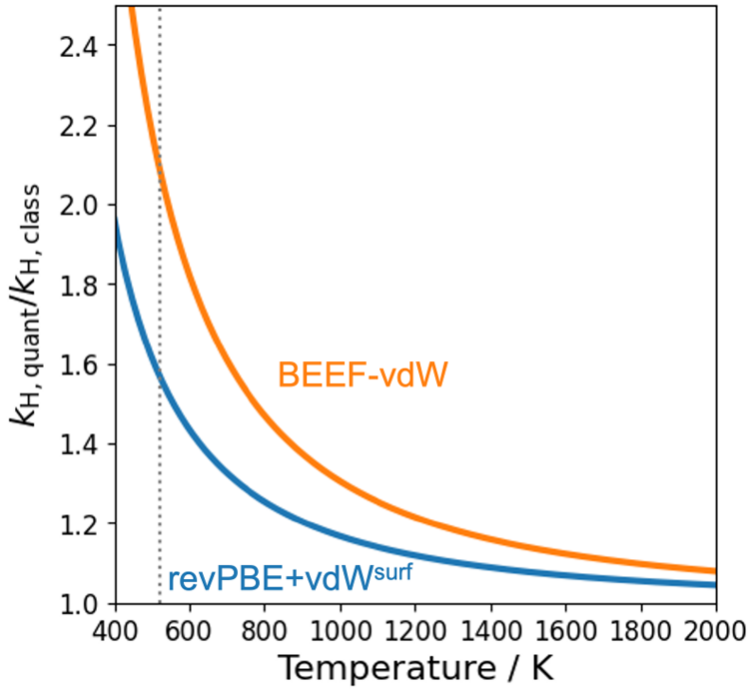
Ratio between harmonic rate constants computed with quantum and classical partition functions. The dotted line marks 523 K, which is the temperature used in the Umbrella Integration simulations.

**Figure 6:**
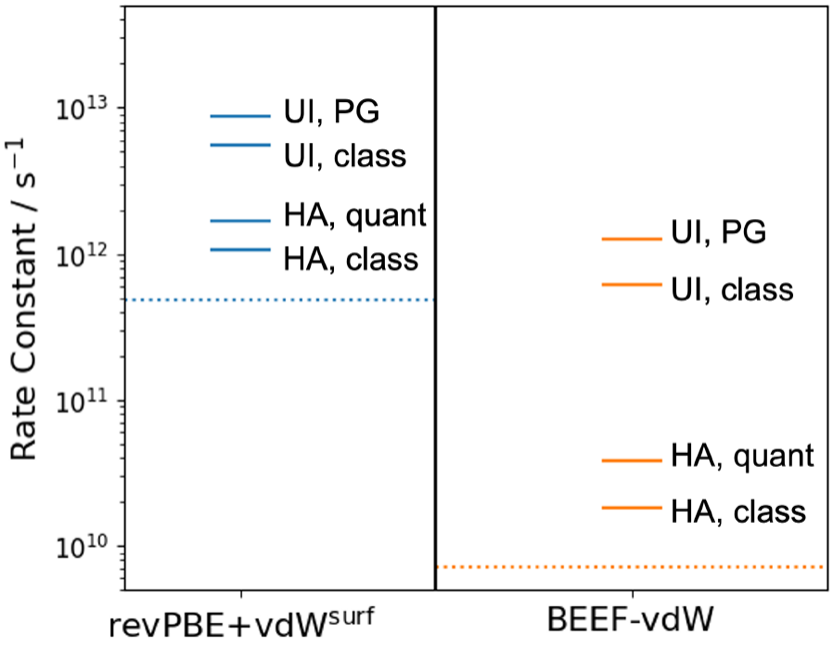
Transition state theory rate constants obtained with the harmonic approximation (HA) using classical and quantum partition functions and using umbrella integration (UI) free energy barriers. In addition to the classical UI rate constants, the influence of quantum nuclear effects can be estimated from the HA using the Pitzer–Gwinn (PG) correction, as detailed in the main text. As a baseline, the dotted lines indicate the TST rate constant when ignoring quantum and thermal effects completely.

We can now compare different approximate rate constants for the decomposition of CHO on Rh(111) (Figure 6). Within the HA, they can be computed by using the classical and quantum partition functions, yielding *k*_H,class_ and *k*_H,quant_. According to the correspondence principle, the classical and quantum models should yield equivalent results in the high-temperature limit. At which temperature this limit is reached in practice depends on the specific vibrational frequencies of the system, however. This is illustrated in Figure 5, where the ratios of the quantum and classical rate constants are plotted as a function of temperature. This reveals that *k*_H,quant_ exceeds *k*_H,class_ by a factor of 1.5 to 2 at 523 K, depending on the DFT functional. While this is a relatively small difference given that rate constants tend to vary by several orders of magnitude in catalytic reaction networks, quantum nuclear effects are clearly not negligible for this reaction.

Next, we can compare the rate constants obtained from the HA with those obtained via the UI free energy barriers (*k*_UI_). These are shown in Figure 6, revealing that the classical, fully anharmonic description of the free energy barrier obtained with UI yields significantly larger rate constants than both the classical and quantum HA. Indeed, the enhancement from *k*_H,class_ to *k*_UI_ is much larger than the enhancement from *k*_H,class_ to *k*_H,quant_ (30 and 5-fold, for BEEF-vdW and revPBE+vdW^surf^, respectively).

Both thermal and quantum effects thus lead to a significant increase in the rate constants relative to the naive baseline of plugging Δ*E* into eq 3 (the dotted lines in Figure 6). To estimate the combined effect of (anharmonic) thermal and quantum effects, the Pitzer–Gwinn (PG) approximation for the partition functions can be used.^41^ To this end, we assume that 
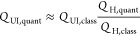
7from which it follows that
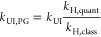
8This leads to estimated rate constants of 1 × 10^12^ s^–1^ (BEEF-vdW) and 9 × 10^12^ s^–1^ (revPBE+vdW^surf^). These rate constants can be understood more intuitively in terms of the half-lives  of CHO they imply. These are 550 and 80 fs for BEEF-vdW and revPBE+vdW^surf^, respectively. Under these conditions, CHO decomposition is thus hardly a rare event.

More generally, it is notable that the inclusion of thermal and nuclear quantum effects changes the rate constants by 1.5 to 2 orders of magnitude, depending on the functional. These effects are thus on the same order of magnitude as the difference between the functionals. Since the potential energy barriers Δ*E* enter eq 4 exponentially while the partition functions are part of the prefactor, the accuracy of DFT transition state energetics has been a prime focus in computational catalysis methods development.^42^ Our results indicate that thermal effects can be equally important for certain reactions.

### III.5 Harmonic Free Energy Corrections

Analogously to the HA for rate constants, it is also possible to define a harmonic free energy approximation by exploiting the equivalence of eq 3 and eq 4. In Figure 4, the HA free energy barriers (based on quantum partition functions) for BEEF-vdW and revPBE+vdW^surf^ are indicated as stars in the central plot. An appealing feature of these harmonic free energy corrections is that they can be decomposed into physically interpretable contributions, namely, the integrated heat capacity *C*_vib_^0→*T*^, the entropic contribution −*TS*_vib_, and the zero point vibrational energy (ZPVE). These contributions to the barrier are shown in Table 1.

**Table 1:** Individual Contributions to Harmonic Free Energy Corrections of the DFT Reaction Barriers at 523 K^a^

Contribution	revPBE+vdW^surf^	BEEF-vdW
*C*_vib_^0→*T*^	–16	–18
–Δ*TS*_vib_	25	16
ΔZPVE	–60	–74
Sum	–51	–76

a The final line is the overall correction. All values are given in meV.

This reveals that the lowering of the HA barriers is mainly due to the ZPVE, which is by far the most negative contribution for both functionals. Meanwhile, the thermal contributions (*C*_vib_^0→*T*^ and −*TS*_vib_) have small and compensating effects on the barrier, with the entropic contributions to the barrier being positive and of similar magnitude to the negative contributions of the integrated heat capacity. This is in stark contrast to the UI predictions, which exclusively cover thermal effects and lead to a much stronger decrease in the barrier.

Indeed, the HA is known to be inadequate for low frequency modes, for which empirical corrections or separate treatments must be used.^12,14^ For this reaction, all real vibrational modes in the initial and transition states display frequencies above 90 cm^–1^, however, so that they would not be considered to be particularly pathological (see SI). Nevertheless, the PES obviously displays considerable anharmonicity. This is likely related to the small reaction barrier and the small geometric changes between the initial and transition states. As a consequence, application of the HA cannot be recommended in such situations.

It should also be noted that HA is remarkably sensitive to small changes in the geometry for this reaction. Specifically, in ref (19), the corresponding free energy barrier is estimated to be 0.49 eV at the BEEF-vdW level (compared to 0.25 eV found herein). In other words, the free energy correction is found to substantially increase the barrier therein, in contrast to our findings at both the UI and HA level. This discrepancy is likely caused by spurious low frequency modes, which disappear when tightly converging the geometry optimization. While such problems can be identified by manual inspection, they are challenging to diagnose in high-throughput settings such as ref (19).

### III.6 Density Functional Comparison

**Figure 7:**
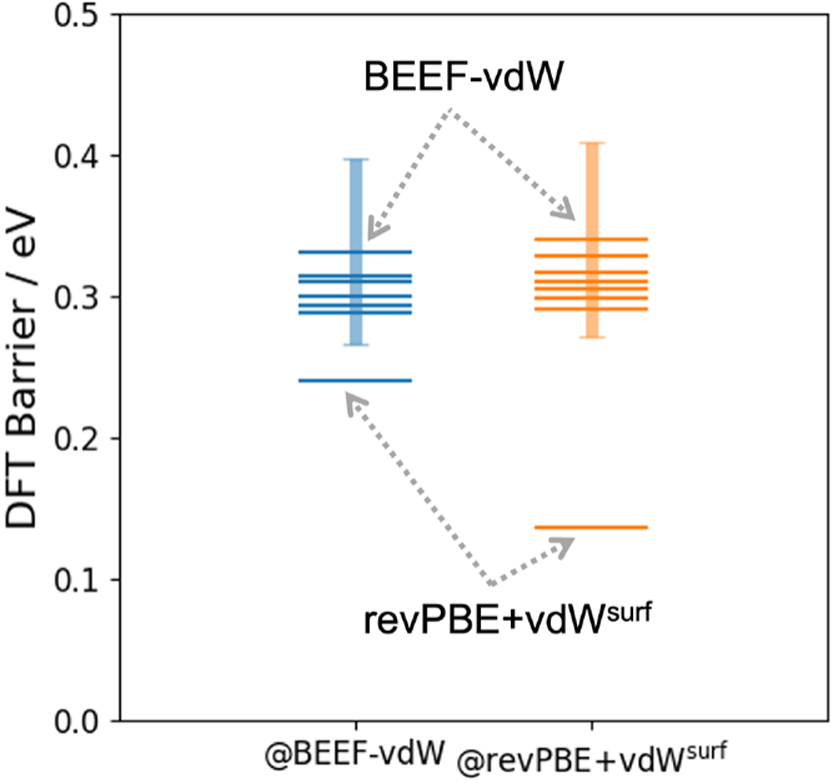
Density functional theory energy barriers obtained with single point calculations using different functionals and dispersion corrections at the BEEF-vdW and revPBE+vdW^surf^ initial and transition state geometries. In both cases, BEEF-vdW and revPBE+vdW^surf^ represent the upper and lower bounds of the estimated barrier, respectively, as indicated by the labels. Details for the other functionals are given in the main text and the SI. The shaded bar indicates the standard deviation of the BEEF ensemble uncertainty estimate.

Figure 6 implies a remarkably large influence of using different dispersion-corrected GGA functionals on the predicted rates. Here, further analysis indicates that this is to some extent an artifact of using the combination of revPBE and the vdW^surf^ dispersion correction. Specifically, the DFT reaction barrier was recomputed using single point calculations with different functionals and dispersion corrections at the BEEF-vdW and revPBE+vdW^surf^ initial and transition state geometries (see Figure 7). Here, we considered the pure PBE and revPBE functionals as well as PBE with both the vdW^surf^ and conventional Tkatchenko–Scheffler dispersion corrections (see the SI for details). This reveals revPBE+vdW^surf^ to be something of an outlier (see the SI for the individual barriers of each functional).

This appears to be related to the fact that revPBE is more repulsive than other GGA functionals like BEEF and PBE, meaning that the onset of the damping function connecting the exchange-correlation functional and the dispersion correction must be set to smaller interatomic distances.^43^ While this works reasonably well for noncovalent interactions in molecular dimers and liquid water,^43^ the performance for adsorbates on surfaces deteriorates.^44^

On the other hand, it is also notable that BEEF-vdW predicts the highest of all barriers, although the other predictions lie within the standard deviation of the BEEF ensemble uncertainty.^37^ Consequently, the BEEF-vdW and revPBE+vdW^surf^ results discussed above represent the upper and lower bounds of the expected barrier within the scope of GGA DFT. Overall, the qualitative behavior of both functionals is analogous, in that the inclusion of thermal effects, anharmonicity, and nuclear quantum effects all lead to a significant increase in the predicted rate constants. In terms of the absolute rates we can conclude that the BEEF-vdW numbers are likely more reliable, though they may be slightly overestimating the barrier.

### III.7 Limitations

The focus of the current article is on obtaining ML potentials for the computation of free-energy profiles (FEPs) in surface catalysis. It should be emphasized, however, that obtaining the FEP itself is not sufficient to obtain the exact rate constants for a given level of theory. There are several further approximations involved in both the computation of the free energy barrier and in TST.

Regarding the former, calculating the free energy barrier from the minimum and maximum of the FEP is an approximation, because the entropy along the CV is neglected. As a consequence, the barrier computed this way is also somewhat dependent on the choice of CV. Dietschreit et al. recently reported formulas for computing exact free energy barriers.^45^ This requires the reconstruction of the reweighted ensemble of all configurations, which is beyond the scope of the current study.

Regarding the use of TST, in particular, the presence of recrossing events can be problematic. A recrossing occurs when a trajectory reaches the transition state from the initial state and subsequently returns to the initial state before it visits the final state. TST assumes that this does not happen, so that the presence of such unsuccessful transitions means that the TST rate constants are too large. This can be addressed by introducing an additional transmission coefficient κ < 1 in eq 3, to account for recrossing.

Given the UI/TST estimated half-lives of 1138 and 124 fs for the two functionals (without the PG correction for nuclear quantum effects), the validity of TST can be tested by running unbiased MD simulations seeded in the initial state. Specifically, when 120 configurations are drawn from MD simulations constrained around the initial state with a biasing potential and subsequently running 2.5 ps long unbiased MD trajectories, reactive events are frequently observed. From these simulations, the half-lives of CHO can empirically be estimated as 725 and 140 fs, respectively. These times are in good agreement with the ones estimated from UI/TST. Indeed, this agreement may be somewhat fortuitous, and it should be noted that the unbiased MD estimates are an imperfect benchmark. In addition to significant statistical uncertainty (see SI), the initial structures are drawn from a biased ensemble and equilibration to the correct initial state distribution is hindered by frequent reactive events.

An alternative approach is to directly estimate the transmission coefficient κ from simulations. To this end, unbiased MD simulations were seeded both at the refined NEB TS and at the maximum of the free energy profile, for the BEEF-vdW based potential. In the latter case, configurations were drawn from restrained MD simulations close to the free energy maximum. In both cases, 240 MD trajectories were started by using 120 random velocities drawn from the Maxwell–Boltzmann distribution and integrating forward and backward in time until either the initial or final state basin is reached (defined as ξ < – 0.2 and ξ > 2.0, respectively).

This yields transmission coefficients of κ = 0.87 and κ = 0.81, respectively. In other words, a moderate amount of recrossing is indeed observed, so that the reported rate constants in Figure 6 are too large by 10–20%. Notably, transmission coefficients on this order to account for recrossing are not uncommon for barrierless reactions in gas-phase kinetics (see, for example, ref (46) and references therein, in which a dynamical correction of 15% was applied).

## IV Conclusions

The current work demonstrates that state-of-the-art free energy calculations can be performed for heterogeneous catalytic reactions with DFT-quality potential energy surfaces. This is achieved by using ML interatomic potentials and a data-efficient iterative training scheme. Overall, the presented approach allows for a more rigorous treatment of thermal effects in computational catalysis and provides a valuable benchmark for approximate free energy corrections.

The present results indicate that CHO is an unstable (or at least extremely short-lived) species on Rh(111). While the formation of this species does not appear to be rate limiting in recent microkinetic simulations of syngas conversion, this finding does have some consequences for the mechanistic understanding of this process. On one hand, the rate limiting step in ref (19) is the subsequent hydrogenation step from CHO to CHOH, which assumes the presence of CHO on the surface. On the other hand, the relative stabilities of CHO and COH determine the selectivity of syngas conversion to different products.

More generally, our results fundamentally call into question the accuracy of the free energy barriers currently used in computational heterogeneous catalysis. We therefore aim to re-examine the reaction network of syngas chemistry on Rh more broadly in future work. In this context, it should be noted that the ML potentials used herein are single-purpose models trained and used for one specific elementary reaction. When treating a full reaction network, there are potentially synergy effects, in the sense that new data for a specific elementary step will likely improve the model for all steps in the network. Similarly, a potential could be trained for different CO and H coverages simultaneously. Indeed, lateral interactions are known to be important for this process.^21^

From a methodological perspective, our analysis furthermore indicates that nuclear quantum effects lead to a further decrease in this reaction barrier. In this context, a more rigorous treatment beyond the PG approximation would be of interest, for example, using path-integral MD^47^ or multicomponent DFT.^48^

## Supporting Information Available

Detailed description of the GAP model hyperparameters, training procedure, vibrational frequencies and density functional differences (PDF)
