# Supplementary material for: Estimating Free Energy Barriers for Heterogeneous Catalytic Reactions with Machine Learning Potentials and Umbrella Integration
Source: J Chem Theory Comput. 2023 Sep 25;19(19):6796–804. doi: 10.1021/acs.jctc.3c00541 (PMC10569033; doi:10.1021/acs.jctc.3c00541)
Supplement: Supplementary file 1 — ct3c00541_si_001.pdf [file ct3c00541_si_001.pdf]

Supporting Information: Estimating Free Energy Barriers for Heterogeneous Catalytic Reactions with Machine Learning Potentials and Umbrella Integration

Sina Stocker[†], Hyunwook Jung[†], Gábor Csányi[‡], C. Franklin Goldsmith[†,§], Karsten Reuter[†], and Johannes T. Margraf[†*]

†Fritz-Haber-Institut der Max-Planck-Gesellschaft, Faradayweg 4-6, 14195 Berlin, Germany. ‡Engineering Laboratory, University of Cambridge, Cambridge CB2 1PZ, United Kingdom. §Brown University, School of Engineering, Providence, RI USA

Hyperparameters

Within the Gaussian approximation potential (GAP) framework, the total energy (E_{tot}) of an atomic configuration with positions $\mathbf{X}_n$ is expressed in terms of local energy contributions fitted by a Gaussian Process. We use here two-body (2b) and many-body (mb) contributions, which are defined as sums of kernel functions ($k(i,j)$)

$$E_{\mathrm{tot}}(\mathbf{X}_n) = \sum_{i,j} \delta_{\mathrm{2b}}^2 \sum_{n=1}^{N_{\mathrm{sparse,2b}}} c_{\mathrm{2b},n} k_{\mathrm{2b}}(r_{ij}, r_n) + \sum_{i,j} \delta_{\mathrm{mb}}^2 \sum_{n=1}^{N_{\mathrm{sparse,mb}}} c_{\mathrm{mb},n} k_{\mathrm{mb}}(\mathbf{p}_i, \mathbf{p}_j). \tag{1}$$

In this context, i and j are atom indices, $r_{ij} \equiv |r_i - r_j|$ represents an interatomic distance between atom i and j within a cutoff radius ($r_{\mathrm{cut,2b}}$) and $\mathbf{p}_i$ is the smooth overlap of atomic position (SOAP) vector of atom i, which describes its atomic environment within the cutoff sphere $r_{\mathrm{cut,SOAP}}$. A cutoff transition width r_Δ defines a region in which the neighborhood density of the SOAP representation is smoothly damped to zero. δ_{2b} and δ_{mb} define weighting factors of the different contributions and represent the standard deviations of the respective Gaussian Processes. $c_{\mathrm{2b},n}$ and $c_{\mathrm{mb},n}$ are the fitting coefficients obtained by minimizing the loss function

$$\mathcal{L} = \sum_{m}^{M} \frac{(y_l - \tilde{y}_l)^2}{\sigma_m^2} + \mathcal{R} \tag{2}$$

in the training, where y_l and $\tilde{y}_l$ are the DFT calculated and predicted quantities (here energies or forces). M is the training set size and $\mathcal{R}$ a regularization term weighted by σ^2. These σ^2 values (σ_{E}^2 for energies and σ_{F}^2 for forces) represent the assumed variance of the errors and can be set globally for the entire training set, per configuration or in case of fitting forces per atom. To accelerate the training procedure, only a set of N_{sparse} representative training points for the 2-body and many-body terms are used, respectively. These points are selected uniformly for the 2-body and based on the CUR algorithm for the many-body contributions .

In our iterative training scheme (see Fig. S5), we heuristically adjust σ_{E} over the iterations so that the potential is more stronglz regularized during earlier iterations where the training set is far from complete. To this end, we start with a value of 0.01 eV and adjust σ_{E} to $\sqrt{\mathrm{RMSE}}$ (for the validation set) if that value is smaller than 0.01 eV (upper threshold) or larger than 0.001 eV (lower threshold). To illustrate this approach, the different validation set $\sqrt{\mathrm{RMSE}}$ of the energies and σ_{E} are both plotted as a function of iterations in Fig. S1.

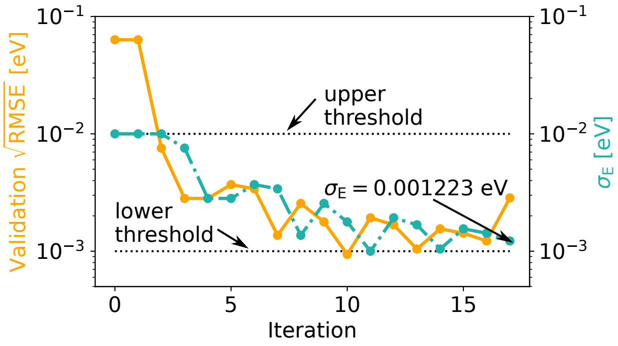

FIG. S1: Energy regularization parameter (σ_{E}) and the square root RMSE of atomization energies per atom of the validation set as a function of iterations. Dotted lines represent the upper and lower threshold of σ_{E}.

We also use different σ_{F} for each atom in the training set. These regularization parameters are adjusted to the DFT calculated forces according to the heuristic formula

$$\sigma_{\mathrm{F},i} = \sigma_{\mathrm{min}} + \frac{C}{A} \log(A \times F_{\mathrm{norm}}^{\mathrm{DFT}} + A \times \overline{F}_{\mathrm{norm}}^{\mathrm{DFT}}). \tag{3}$$

Here, the idea is to incorporate both the DFT force norm of each atom ($F_{\mathrm{norm}}^{\mathrm{DFT}}$) and the average force norm in each configuration ($\overline{F}_{\mathrm{norm}}^{\mathrm{DFT}}$) into the regularization parameter. The parameters σ_{min}, C and A are additional hyperparameters, which are set to $\sigma_{\mathrm{min}} = C = \sqrt{\sigma_{\mathrm{E}}}$ and $A = 0.01$, while different values are used for dimer configurations ($\sigma_{\mathrm{min}} = C = 0.1$). The force regularization parameters are plotted as a function of the force norms in Fig. S2. Note

that the force MAE stated in the main text represent MAEs weighted by the individual σ_{F}:

$$\mathrm{Force\,MAE} \equiv \frac{\sum_i w_i |F_i^{\mathrm{DFT}} - F_i^{\mathrm{GAP}}|}{\sum_i w_i}, \tag{4}$$

with $w_i = \frac{1}{\sigma_{\mathrm{F},i}}$. In addition, we only incorporate forces of the surface atoms into the training evaluation (the CHO

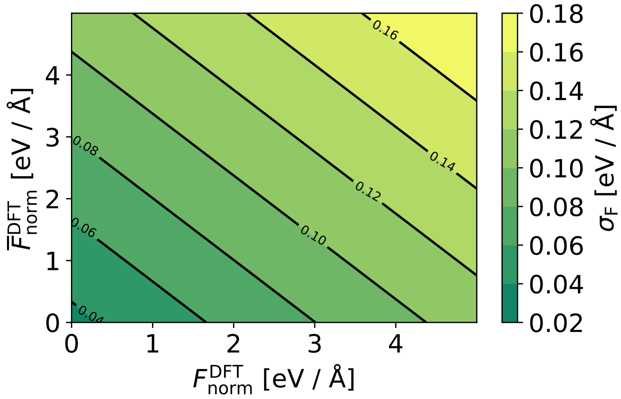

FIG. S2: Force regularization parameter (σ_{F}) as a function of the force norm on an atom (F_{norm}^{DFT}) and the average force norm on a configuration ($\overline{F}_{\mathrm{norm}}^{\mathrm{DFT}}$) .

molecule plus the upper layer of Rh) and mask the remaining Rh-atoms (three bottom layers). This helps to decrease the force MAEs of carbon, oxygen and hydrogen as the surface slab is dominated by Rh-atoms ($\mathrm{Rh} : \mathrm{C} + \mathrm{H} + \mathrm{O} = 36 : 1 + 1 + 1$). Figure S3 indicates the surface and masked atoms.

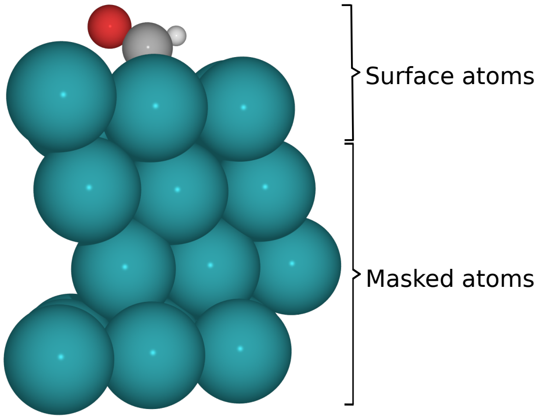

FIG. S3: Illustration of surface and masked atoms during training.

To describe the short and medium range contributions in an atomic environment differently, we use a multi-SOAP approach for the light species (C, H, O). This means that two SOAP representations with different length scale parameters are applied to obtain a comprehensive description of the atoms that are directly involved in the reaction. In contrast, the Rh-atoms are described by a single SOAP lengthscale. In the following, we will distinguish the representations for the light elements by using the notation SOAP_1 as well as SOAP_2 and $\mathrm{SOAP}_{\mathrm{Rh}}$ for the Rh-atoms.

We use a Gaussian kernel function to express the similarity between the 2-body contributions

$$k_{2\mathrm{b}}(r_{ij}, r_n) = \exp\left(-\frac{1}{2}\frac{|r_{ij} - r_n|^2}{\theta^2}\right) \tag{5}$$

and a polynomial kernel

$$k_{\mathrm{mb}}(\mathbf{p}_i, \mathbf{p}_j) = (\mathbf{p}_i \cdot \mathbf{p}_j)^{\zeta} \tag{6}$$

for the many-body contributions. Table S1 lists all relevant hyperparameters herein.

**Hyperparameter**	**2-body**	**SOAP$_1$**	**SOAP$_2$**	**SOAP$_{\mathrm{Rh}}$**
r_{cut} [Å]	3.5	3	5	6
r_{Δ} [Å]	0.5	0.5	0.8	1.0
δ [eV]	2	0.3	0.3	0.3
N_{sparse}	50	2000	2000	1000
θ [Å]	1.0	—	—	—
σ_{at} [Å]	—	0.3	0.5	0.6
ζ	—	4	4	4
b [eV]	—	0	0	0
n_{max}	—	9	9	9
l_{max}	—	3	3	3

TABLE S1: Used hyperparameters.

To avoid unphysical atomic clashes in early training iterations (where the potential is not necessarily fully robust), we add simple diatomic baseline potentials for all element combinations, as reported in [1]. The corresponding O − O potential is plotted in Fig. S4 as an example. These baseline potentials account for the repulsive nature at small interatomic distances and keep structures away from nonphysical configurations in dynamical simulations. If no baseline potential is used, configurations with small distances have to be provided in the training set.

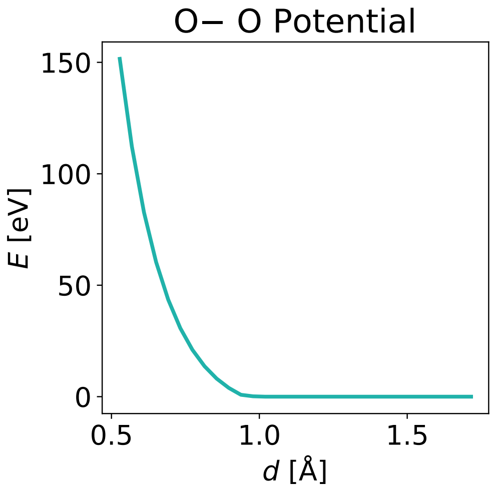

FIG. S4: Example O − O baseline potential as a function of the interatomic distance from [1].

Iterative Training

We start with an initial training set of 50 configurations including 10 structures of the stoichiometry $Rh_{36}CHO$ (namely the NEB trajectory in Fig. 2 main text) and 12 structures of stoichiometry Rh_{36} with an optimized lattice constant of 3.85 Å. In addition, we add some configurations which are computational efficient to evaluate, namely the four isolated atoms (Rh, C, O, H) and 24 dimer combinations of the three light species in gas-phase. With this initial training set, we train a first GAP model (iteration 0). At this stage, geometry optimizations on biased potential energy surfaces in ten randomly selected umbrellas were performed. The energies and forces of these 10 generated structures were recalculated with DFT and the corresponding configurations added to the training set. In subsequent iterations, 2.5 ps of biased dynamics at 573 K (umbrella sampling with k = 15) were carried out, again

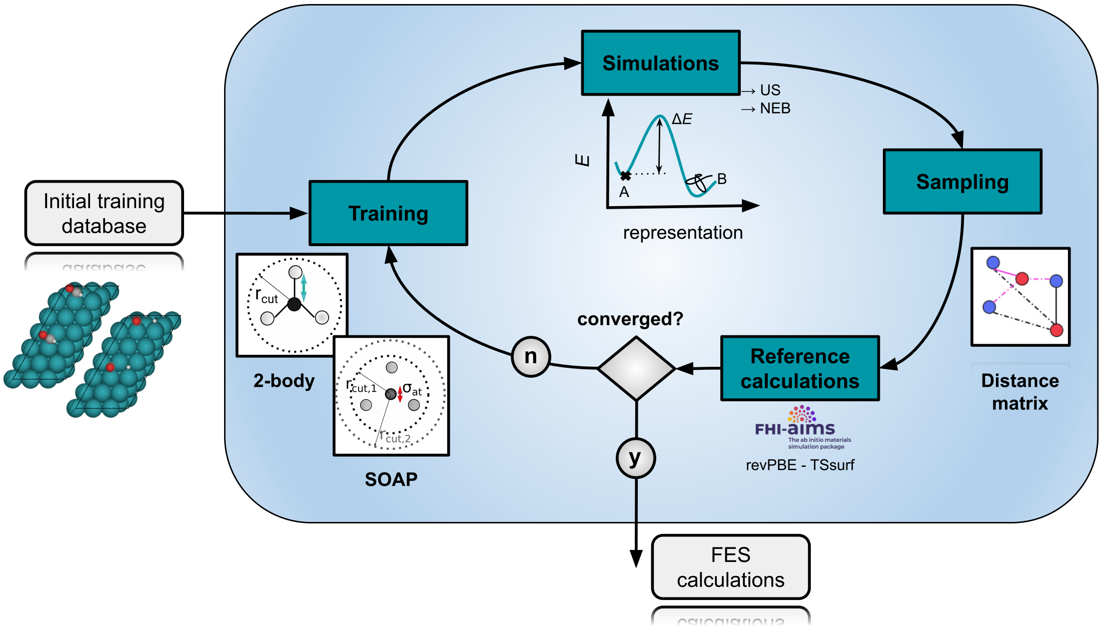

FIG. S5: Iterative training workflow for fitting GAP models. Our approach is explained in the main text of the SI.

in 10 randomly selected windows. From each of these trajectories, one configuration was selected by farthest point sampling (FPS)[2] and added to the training set. After 12 iterations, we additionally perform NEB calculations and further add these images (8 configurations between initial and final state) to the training set. Note, that we rejected structures with force components higher than 15 eV/Å . The workflow is illustrated in Fig. S5. We stop our iterations after 17 iterations and validate the GAP model by monitoring the energy and force errors over the different iterations (see Fig. 3 main text) as well as by comparing NEB trajectories produced by the GAP model and DFT (see Fig. S6). The final training set contains 292 configurations and the potentials were trained on DFT forces as well as atomization energies (AE)

$$\mathrm{AE} = E_{\mathrm{slab}} - \sum_{s}^{N_{\mathrm{atoms}}} E_{\mathrm{atom},s}, \tag{7}$$

where E_{slab} represents the potential energy of the surface slab and E_{atom} the energy of the isolated atom s.

gap_fit command

```
gap_fit default_sigma={0.001222685832523628 0 0 0} energy_parameter_name=dft_energy
force_parameter_name=dft_forces force_mask_parameter_name=force_mask
do_copy_at_file=F sparse_separate_file=F gp_file=GAP.xml at_file=train_revPBE-TSsurf.xyz
core_param_file=glue_revPBE-TSsurf.xml core_ip_args={IP Glue}
gap={distance_Nb order=2 cutoff=3.5 delta=2.0 covariance_type=ard_se n_sparse=50
theta_uniform=1.0 sparse_method=uniform add_species=T :soap cutoff=3 atom_sigma=0.3
l_max=3 n_max=9 covariance_type=dot_product zeta=4 add_species=F n_sparse=2000
sparse_method=CUR_POINTS delta=0.3 n_species=4 Z=1 species_Z={{6 8 45 1}} :
soap cutoff=5 atom_sigma=0.5 l_max=3 n_max=9 covariance_type=dot_product zeta=4
add_species=F n_sparse=2000 sparse_method=CUR_POINTS cutoff_transition_width=0.8
delta=0.3 n_species=4 Z=1 species_Z={{6 8 45 1}} :soap cutoff=3 atom_sigma=0.3
```

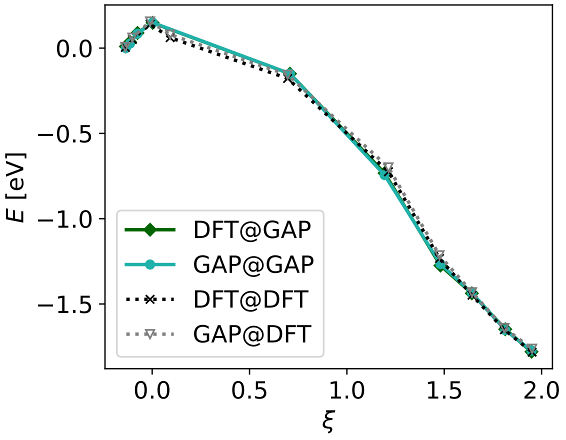

FIG. S6: Model validation by comparing NEB trajectories produced with the GAP model (GAP@GAP) and DFT (DFT@DFT). In addition, the energies of the NEB images generated with the GAP model have been recalculated with DFT (DFT@GAP) and vice versa (GAP@DFT).

```
l_max=3 n_max=9 covariance_type=dot_product zeta=4 add_species=F n_sparse=2000
sparse_method=CUR_POINTS delta=0.3 n_species=4 Z=6 species_Z={{6 8 45 1}} :
soap cutoff=5 atom_sigma=0.5 l_max=3 n_max=9 covariance_type=dot_product zeta=4
add_species=F n_sparse=2000 sparse_method=CUR_POINTS cutoff_transition_width=0.8
delta=0.3 n_species=4 Z=6 species_Z={{6 8 45 1}} :soap cutoff=3 atom_sigma=0.3
l_max=3 n_max=9 covariance_type=dot_product zeta=4 add_species=F n_sparse=2000
sparse_method=CUR_POINTS delta=0.3 n_species=4 Z=8 species_Z={{6 8 45 1}} :
soap cutoff=5 atom_sigma=0.5 l_max=3 n_max=9 covariance_type=dot_product zeta=4
add_species=F n_sparse=2000 sparse_method=CUR_POINTS cutoff_transition_width=0.8
delta=0.3 n_species=4 Z=8 species_Z={{6 8 45 1}} :soap cutoff=6 atom_sigma=0.6
l_max=3 n_max=9 covariance_type=dot_product zeta=4 add_species=F n_sparse=1000
sparse_method=CUR_POINTS cutoff_transition_width=1 delta=0.3 n_species=4 Z=45
species_Z={{6 8 45 1}}}
```

BEEF-vdW Model

The BEEF-vdW model was trained on a subset of the configurations used for the revPBE models, where energies and forces where recomputed with the BEEF-vdW functional. Here, for technical reasons only the configurations inculding the full surface adsorbate system (with composition $Rh_{36}CHO$) where used. This is because isolated atoms and dimer configurations are more challenging to converge with the plane-wave QE code. Since these configurations are mainly included to provide robust prior information about interatomic interactions in early iterations, they can be neglected when retraining on the full revPBE training set. For similar reasons, formation energies were used as the fitting target, defined as:

$$E_{\mathrm{form}} = E_{\mathrm{slab}} - \sum_{s}^{N_{\mathrm{atoms}}} \mu_s, \qquad (8)$$

with $\mu_{\mathrm{H}} = \frac{1}{2}E_{\mathrm{H_2}}$, $\mu_{\mathrm{O}} = E_{\mathrm{H_2O}} - 2\mu_{\mathrm{H}}$, $\mu_{\mathrm{C}} = E_{\mathrm{CO}} - \mu_{\mathrm{O}}$ and $\mu_{\mathrm{Rh}} = \frac{1}{36}E_{\mathrm{Rh_{36}}}$.

Identical hyperparameters as in the last iteration of the revPBE potential are used, merely adjusting σ_{F} according to the BEEF-vdW forces.

gap_fit command

```
gap_fit default_sigma={0.001222685832523628 0 0 0} energy_parameter_name=E_form
force_parameter_name=dft_forces force_mask_parameter_name=force_mask
do_copy_at_file=F sparse_separate_file=F gp_file=GAP.xml
at_file=train_BEEF-vdW.xyz core_param_file=glue_BEEF-vdW.xml core_ip_args={IP Glue}
e0={H:0.0:C:0.0:O:0.0:Rh:0.0} gap={distance_Nb order=2 cutoff=3.5 delta=2.0
covariance_type=ard_se n_sparse=50 theta_uniform=1.0 sparse_method=uniform Z={{45 45 }}:
distance_Nb order=2 cutoff=3.5 delta=2.0 covariance_type=ard_se n_sparse=50
theta_uniform=1.0 sparse_method=uniform Z={{45 1 }} :distance_Nb order=2 cutoff=3.5
delta=2.0 covariance_type=ard_se n_sparse=50 theta_uniform=1.0 sparse_method=uniform
Z={{45 6 }} :distance_Nb order=2 cutoff=3.5 delta=2.0 covariance_type=ard_se
n_sparse=50 theta_uniform=1.0 sparse_method=uniform Z={{45 8 }} :distance_Nb order=2
cutoff=3.5 delta=2.0 covariance_type=ard_se n_sparse=50 theta_uniform=1.0
sparse_method=uniform Z={{1 6 }} :distance_Nb order=2 cutoff=3.5 delta=2.0
covariance_type=ard_se n_sparse=50 theta_uniform=1.0 sparse_method=uniform Z={{1 8 }}
:distance_Nb order=2 cutoff=3.5 delta=2.0 covariance_type=ard_se n_sparse=50
theta_uniform=1.0 sparse_method=uniform Z={{6 8 }} :soap cutoff=3 atom_sigma=0.3
l_max=3 n_max=9 covariance_type=dot_product zeta=4 add_species=F n_sparse=2000
sparse_method=CUR_POINTS delta=0.3 n_species=4 Z=1 species_Z={{6 8 45 1}} :soap
cutoff=5 atom_sigma=0.5 l_max=3 n_max=9 covariance_type=dot_product zeta=4
add_species=F n_sparse=2000 sparse_method=CUR_POINTS cutoff_transition_width=0.8
delta=0.3 n_species=4 Z=1 species_Z={{6 8 45 1}} :soap cutoff=3 atom_sigma=0.3
l_max=3 n_max=9 covariance_type=dot_product zeta=4 add_species=F n_sparse=2000
sparse_method=CUR_POINTS delta=0.3 n_species=4 Z=6 species_Z={{6 8 45 1}} :soap
cutoff=5 atom_sigma=0.5 l_max=3 n_max=9 covariance_type=dot_product zeta=4
add_species=F n_sparse=2000 sparse_method=CUR_POINTS
cutoff_transition_width=0.8 delta=0.3 n_species=4 Z=6 species_Z={{6 8 45 1}} :soap
cutoff=3 atom_sigma=0.3 l_max=3 n_max=9 covariance_type=dot_product zeta=4
add_species=F n_sparse=2000 sparse_method=CUR_POINTS delta=0.3 n_species=4 Z=8
species_Z={{6 8 45 1}} :soap cutoff=5 atom_sigma=0.5 l_max=3 n_max=9
covariance_type=dot_product zeta=4 add_species=F n_sparse=2000
sparse_method=CUR_POINTS cutoff_transition_width=0.8 delta=0.3 n_species=4 Z=8
species_Z={{6 8 45 1}} :soap cutoff=6 atom_sigma=0.6 l_max=3 n_max=9
covariance_type=dot_product zeta=4 add_species=F n_sparse=1000
sparse_method=CUR_POINTS cutoff_transition_width=1 delta=0.3 n_species=4 Z=45
species_Z={{6 8 45 1}}}
```

Initial State	Transition State
175.5	170.9
210.8	232.5
451.8	420.9
453.4	500.7
596.6	553.6
647.6	1050.4
1187.0	1295.5
1200.5	2645.9
2937.3	

TABLE S2: Vibrational frequencies of initial and transition states at the revPBE+vdW^{surf} level in cm^{-1}.

* margraf@fhi.mpg.de

Initial State	Transition State
151.4	94.30
201.5	220.0
255.4	264.8
349.1	389.7
496.3	550.2
662.1	1098.4
1180.5	1494.0
1276.0	2188.7
2927.5	

TABLE S3: Vibrational frequencies of initial and transition states at the BEEF-vdW level in cm^{-1}.

Code	Functional	ΔE@BEEF-vdW	ΔE@revPBE+vdW^{surf}
FHI-aims	revPBE+vdW^{surf}	0.24	0.14
FHI-aims	revPBE	0.31	0.32
FHI-aims	PBE	0.31	0.31
FHI-aims	PBE+vdW^{surf}	0.31	0.31
FHI-aims	PBE+TS	0.29	0.29
Quantum Espresso	BEEF-vdW	0.33	0.34
Quantum Espresso	PBE	0.29	0.30
Quantum Espresso	revPBE	0.30	0.32

TABLE S4: Energy barriers (in eV) computed with different codes and functionals using the BEEF-vdW and revPBE+vdW^{surf} initial and transition state geometries.

[1] H. Jung, L. Sauerland, S. Stocker, K. Reuter, and J. T. Margraf, ChemRxiv (2022), https://doi.org/10.26434/chemrxiv-2022-q3j0s.

[2] S. De, A. P. Bartók, G. Csányi, and M. Ceriotti, Phys. Chem. Chem. Phys. **18**, 13754 (2016).

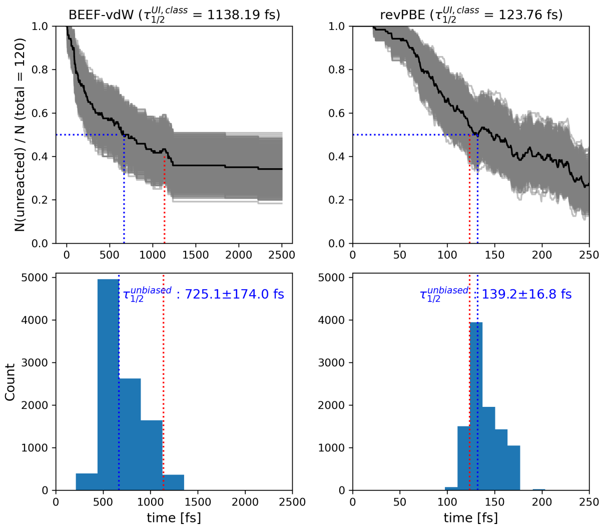

FIG. S7: Estimation of CHO half-lives from unbiased MD simulations. Statistical errors are estimated via bootstrapping.
